# Supplementary figures and images for: Orienting attention to auditory and visual working memory in older adults with cochlear implants
Source: PLoS One. 2024 Dec 26;19(12):e0310082. doi: 10.1371/journal.pone.0310082 (PMC11670960; doi:10.1371/journal.pone.0310082)

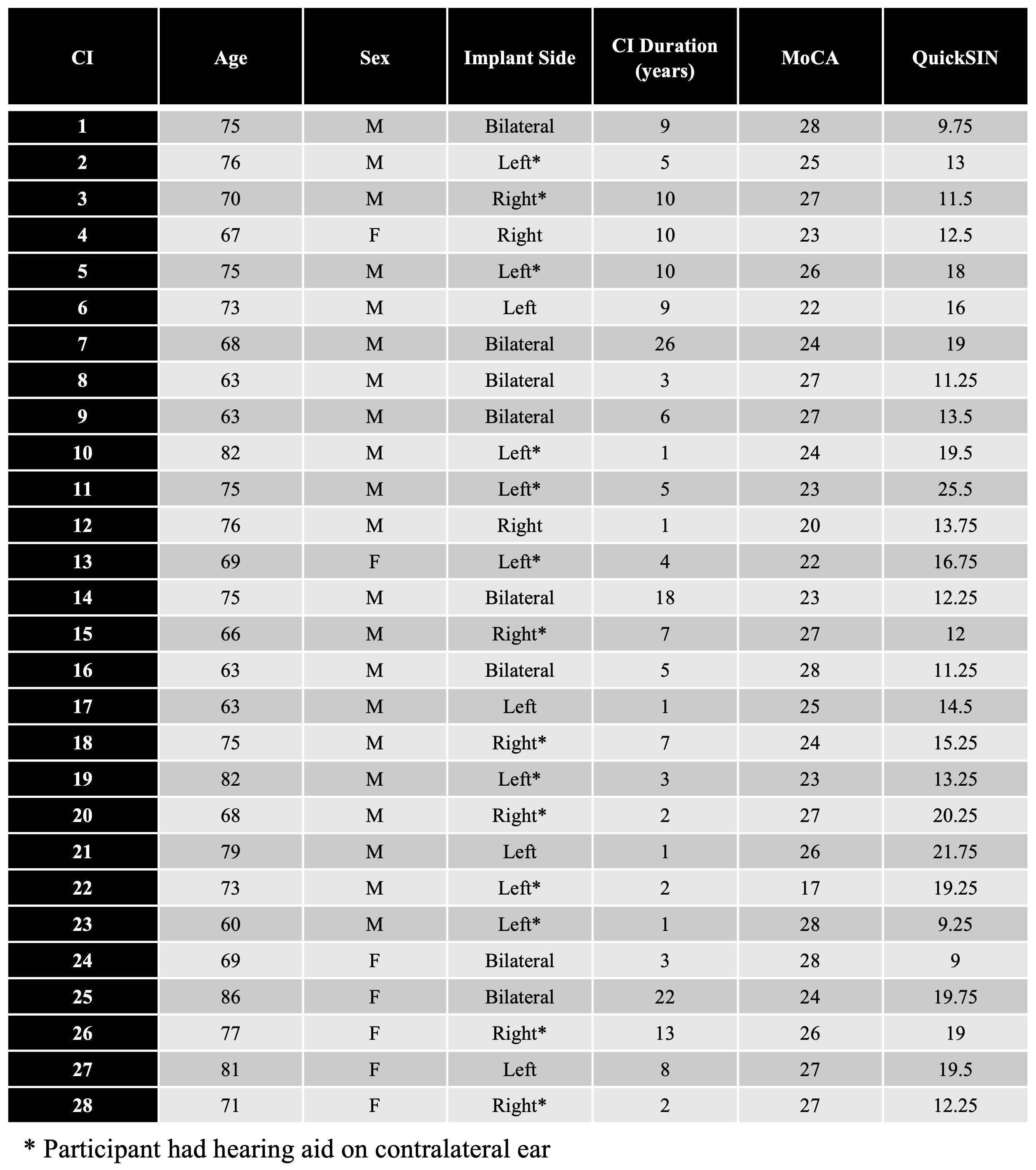

Supplement: S1 Table — (TIFF) [file pone.0310082.s001.tiff]

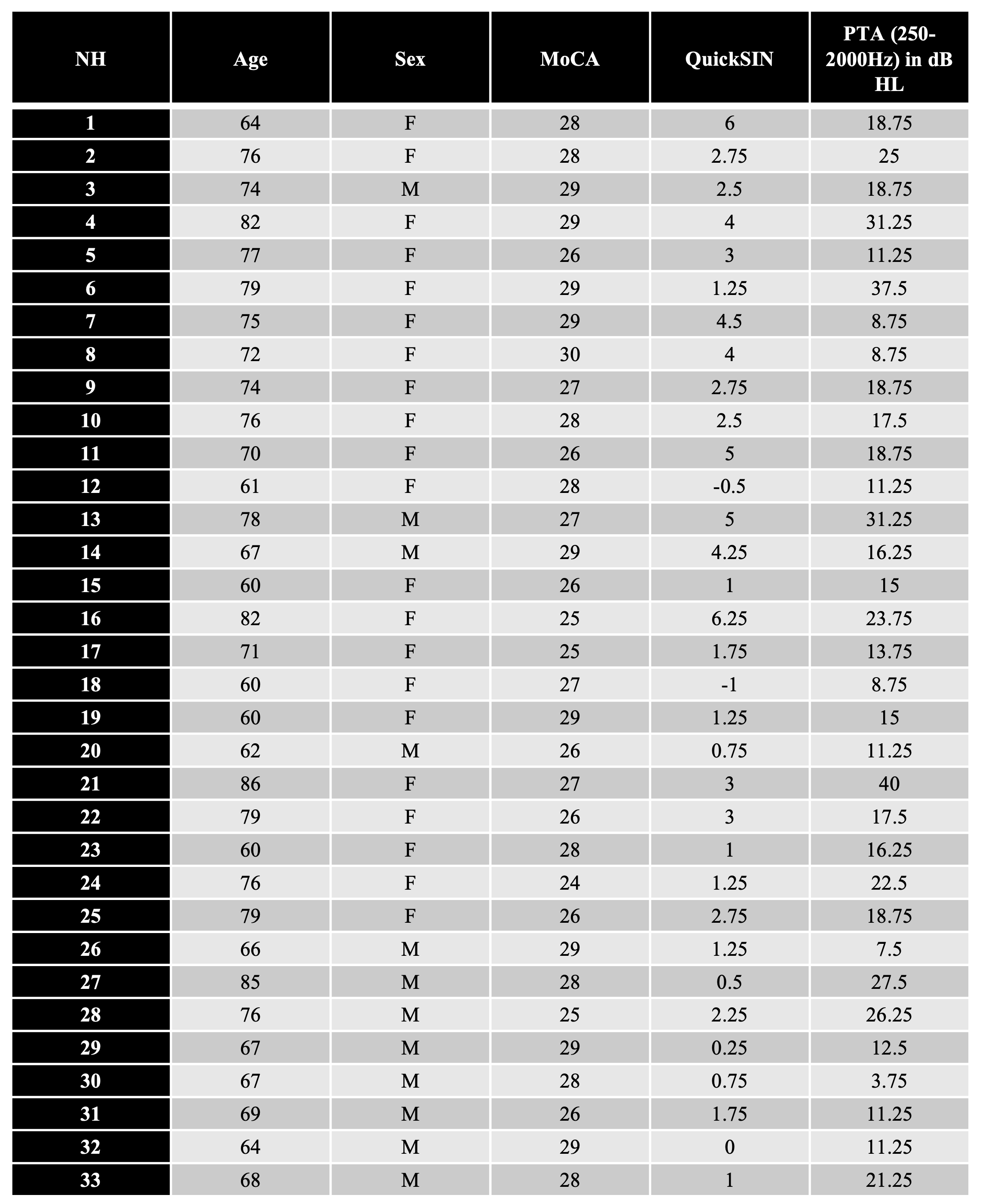

Supplement: S2 Table — (TIFF) [file pone.0310082.s002.tiff]
