# Supplementary material for: Orienting attention to auditory and visual working memory in older adults with cochlear implants
Source: PLoS One. 2024 Dec 26;19(12):e0310082. doi: 10.1371/journal.pone.0310082 (PMC11670960; doi:10.1371/journal.pone.0310082)
Supplement: Attachment — Submitted filename: PlosOne2024Feb14Review.pdf [file pone.0310082.s004.pdf]

The study investigates the cue orientation and reflective attention performance of cochlear implant (CI) users within a working memory (WM) task compared to healthy controls. They show that Although in general auditory working memory performance the control group are significantly better than the CI group, there is no significant difference between groups for the retro-cue performance for either auditory or visual WM. Their results suggest that CI users are able to orient their memory similar to typical hearing age matched adults.

The introduction was clear with well explained. The Methods partn needs a bit more details but in general well explained. Results explained in detail clearly. Discussion was read well. I have some comments below:

Methods

Experimental procedure (pp 136-141): *Prior to beginning the experiment, participants completed different versions of unimodal (auditory and visual) delayed match-to-sample tasks to familiarize the participants with the paradigm and to adjust the level of discriminability*

What is the number of participants in each group taking which version of the unimodal tasks? Were the number of participants taking each unimodal task balanced for each group?

pp 148-151: *The physical difference between the gradients (i.e., visual angle rotation) and piano tone (i.e., frequency) varied between versions of the tasks. The difference in visual angle between gradients ranged from a random rotation of 25 to 60 degrees for the visual delayed match-to sample task. For the auditory delayed match-to-sample, the difference in frequency varied from one-quarter tone to seven semitones.*

Are these differences counterbalanced within participant? How many trials were there for this task? Were the total number of trials for these tasks same for each participant? Otherwise, there might be a learning effect confound. Are these tasks used a version of a JND to equate the difficulty of the main task for each participant? Is so, are both tasks performed by all participants? This part is unclear in the methods section.

pp. 165-170: *Once the stimuli disappeared, a black screen was shown for 1000ms, followed by another 500ms presentation of a letter retro-cue which was either neutral (X) or informative (A or V to indicate whether to orient to auditory or visual modality, respectively). In trials with the informative A retro-cue, participants were tasked to remember the piano tone, whereas trials with the informative V retro-cue, participants were instructed to remember the visual gradients.*

What is the instruction for neutral (X) trials?

p 170: .... *a test item was presented for 500ms, and participants indicated whether the item matched with the auditory*

Is the test item unimodal? What is the probe for each condition? In neutral condition, if the probe is bimodal, what are ‘different’ probe conditions (always both sensory modalities different?)

Results

Does the duration of CI usage have an effect on the WM performance?

Discussion

The unimodal DMTS task suggests a possibility of discrimination problem instead of a WM performance difference in the CI users. This should be discussed a bit more in the paper.
