# Supplementary material for: Orienting attention to auditory and visual working memory in older adults with cochlear implants
Source: PLoS One. 2024 Dec 26;19(12):e0310082. doi: 10.1371/journal.pone.0310082 (PMC11670960; doi:10.1371/journal.pone.0310082)
Supplement: Attachment — Submitted filename: Response to Reviewers.docx [file pone.0310082.s006.docx]

We thank the two reviewers for their constructive comments and insightful feedback. Below is our point-by-point response to each comment.

**Reviewer #1:**

*The task for the uninformative (X) condition is still unclear in the methods section. From what I gathered, they needed to remember both A and V items and respond to a unilateral item but it is still not clear. As this is 50% of the trials. It would be a good idea to clarify. If this is not the case, the Audiovisual delayed match to sample explanation still needs more clarification.*

RESPONSE: We thank the reviewer for highlighting this point. The reviewer's interpretation is correct. We have revised the methods section to clarify the description of the uninformative (X) condition, which now reads as follows:

“In the neutral X retro-cue condition, participants were required to retain both auditory and visual items in memory, as no indication was provided regarding which modality would be tested. The test item could originate from either modality, necessitating participants to match it with the corresponding item in their working memory. This condition accounted for 50% of the trials and required participants to be prepared for either test item.”
